# Supplementary material for: Synergetic Effects of Intronic Mature miR-944 and ΔNp63 Isoforms on Tumorigenesis in a Cervical Cancer Cell Line
Source: Int J Mol Sci. 2020 Aug 5;21(16):5612. doi: 10.3390/ijms21165612 (PMC7460632; doi:10.3390/ijms21165612)
Supplement: Supplementary file 1 [file ijms-21-05612-s001.zip › Supplementary conversion/ijms-865581 supplementary Table S1-3 conversion.docx]

Supplementary Materials

**Table S1.** List of GO terms by functional analysis.

| **Condition** | **Cluster** | **GO Terms** | **No. of Genes** | **%** | ***p*-value** |
| --- | --- | --- | --- | --- | --- |
| *miR-944*  inhibition | 1 | GO:0008219~cell death | 4 | 0.014 | <0.05 |
|  | 2 | GO:0005913~cell-cell adherens junction | 8 | 0.029 | <0.05 |
|  | 3 | GO:0006334~nucleosome assembly | 5 | 0.018 | <0.05 |
| *ΔNp63*  Inhibition | 1 | GO:0042981~regulation of apoptotic process | 16 | 0.029 | <0.001 |
|  | 2 | GO:0030855~epithelial cell differentiation | 8 | 0.014 | <0.001 |
|  |  | GO:0008544~epidermis development | 8 | 0.014 | <0.05 |
|  |  | GO:0005923~bicellular tight junction | 7 | 0.013 | <0.05 |
|  |  | GO:0048468~cell development | 4 | 0.007 | <0.05 |
|  | 3 | GO:0007165~signal transduction | 37 | 0.067 | <0.05 |
|  |  | GO:0051091~positive regulation of sequence-specific DNA binding transcription factor activity | 8 | 0.014 | <0.05 |
|  |  | GO:0043565~sequence-specific DNA binding | 20 | 0.036 | <0.05 |
|  | 4 | GO:0009615~response to virus | 6 | 0.011 | <0.05 |
| *miR-944*  + *ΔNp63*  inhibition | 1 | GO:0008285~negative regulation of cell proliferation | 42 | 0.028 | <0.001 |
|  |  | GO:0043065~positive regulation of apoptotic process | 34 | 0.022 | <0.001 |
|  |  | GO:0045926~negative regulation of growth | 6 | 0.004 | <0.05 |
|  | 2 | GO:0008544~epidermis development | 17 | 0.011 | <0.001 |
|  |  | GO:2000145~regulation of cell motility | 7 | 0.005 | <0.05 |
|  |  | GO:0042060~wound healing | 12 | 0.008 | <0.05 |
|  |  | GO:0007267~cell-cell signaling | 26 | 0.017 | <0.05 |
|  |  | GO:0030334~regulation of cell migration | 11 | 0.007 | <0.05 |
|  | 3 | GO:0045944~positive regulation of transcription from RNA polymerase II promoter | 97 | 0.064 | <0.001 |
|  |  | GO:0006335~DNA replication-dependent nucleosome assembly | 12 | 0.008 | <0.001 |
|  |  | GO:0045815~positive regulation of gene expression, epigenetic | 16 | 0.011 | <0.001 |
|  |  | GO:0045814~negative regulation of gene expression, epigenetic | 14 | 0.009 | <0.001 |
|  |  | GO:0060968~regulation of gene silencing | 7 | 0.005 | <0.001 |
|  |  | GO:0031047~gene silencing by RNA | 14 | 0.009 | <0.05 |
|  | 4 | GO:0060337~type I interferon signaling pathway | 15 | 0.010 | <0.001 |
|  |  | GO:0051607~defense response to virus | 22 | 0.014 | <0.001 |
|  |  | GO:0002227~innate immune response in mucosa | 7 | 0.005 | <0.05 |
|  |  | GO:0009615~response to virus | 15 | 0.010 | <0.05 |

**Table S2.** Primers, probes, and siRNA sequences of *p63*.

| **Target gene** | | **Sequence (5′-3′)** | **PCR product (bp)** | **Annealing temperature**  **(℃)** |
| --- | --- | --- | --- | --- |
| *TAp63* | F ^a^ | TGT ATC CGC ATG CAG GAC T |  |  |
|  | R ^b^ | CTG TGT TAT AGG GAC TGG TGG AC | 127 | 55 |
|  | P ^c^ | FAM-TCC TGA ACA GCA TGG ACC AGC A-BHQ1 |  |  |
| *ΔNp63* | F | AGT GAG CCA CAG TAC ACG |  |  |
|  | R | CCT GAA CAG CAT GGA CCA GCA G | 97 | 55 |
|  | P | FAM-CCC TAT AAC ACA GAC CAC G-BHQ1 |  |  |
|  | siRNA | Sense: GGA CAG CAG CAU UGA UCA A |  |  |
|  |  | Antisense: UUG AUC AAU GCU GCU GUC C |  |  |
| *GAPDH* | F | CCA TCT TCC AGG AGC GAG ATC C |  |  |
|  | R | ATG GTG GTG AAG ACG CCA GTG | 90 | 55 |
|  | P | FAM-TCC ACG ACG TAC TCA GCG CCA GCA-BHQ1 |  |  |

a Forward primer; b Reverse primer; c Probe

**Table S3.** Antibodies used for the analysis of protein expression in ME-180 cells.

| **Target** | **MW (kDa)** | **Source of secondary antibody** | **Dilution ratio** | **Company** |
| --- | --- | --- | --- | --- |
| E-cadherin | 135 | rabbit | 1:3000 | Cell Signaling |
| N-cadherin | 140 | rabbit | 1:3000 | Cell Signaling |
| Slug | 30 | rabbit | 1:3000 | Cell Signaling |
| Vimentin | 57 | rabbit | 1:1000 | Cell Signaling |
| Claudin | 20 | rabbit | 1:3000 | Cell Signaling |
| cleaved PARP | 89 | rabbit | 1:3000 | Cell Signaling |
| β-actin | 45 | mouse | 1:5000 | Santacruz |


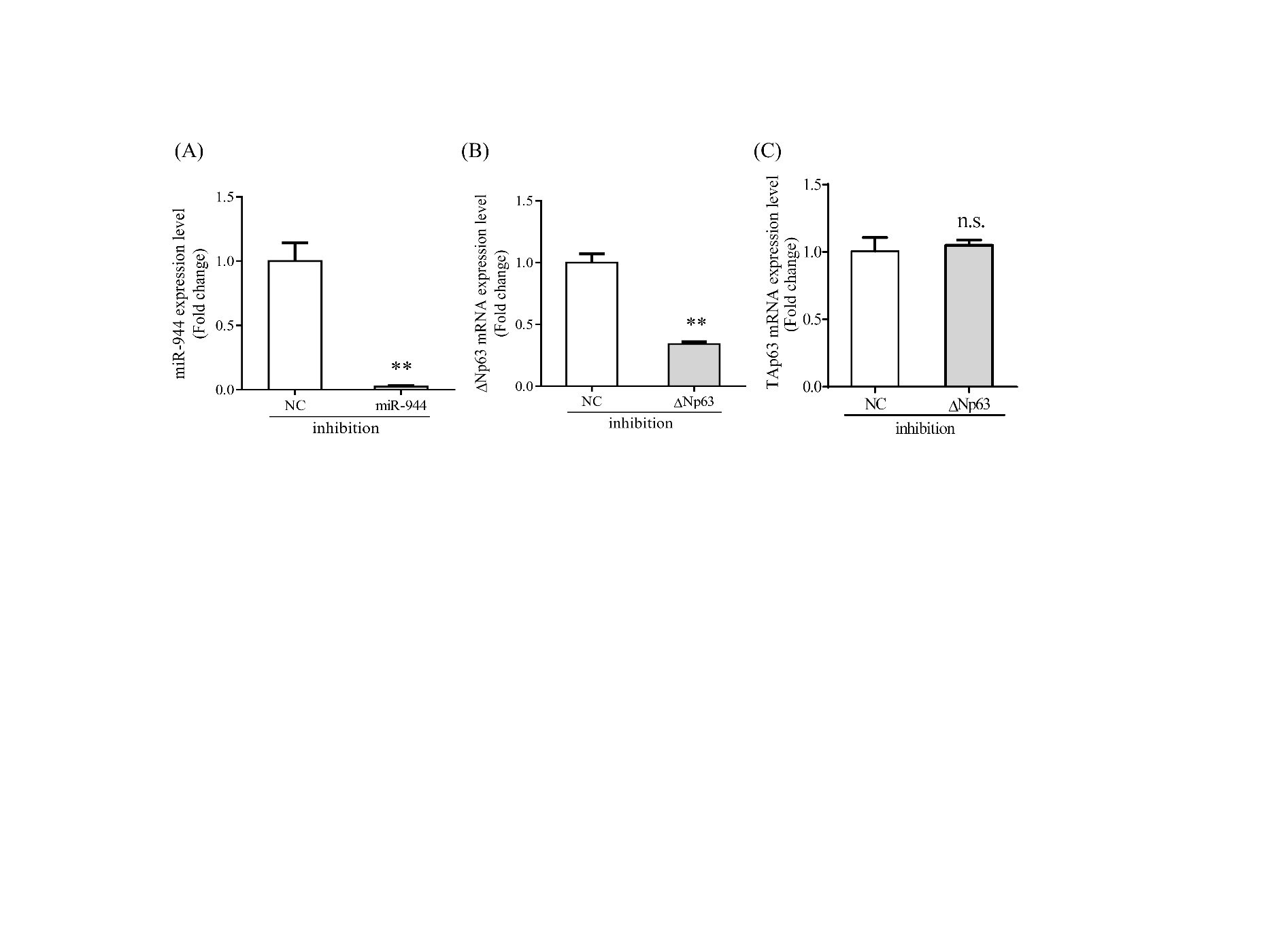


**Figure S1.** Inhibitory effects by *miR-944* and *ΔNp63* inhibition. The expression levels of (**A**) *miR-944*, (**B**) *ΔNp63*, and (**C**) *TAp63* after transfection of anti-miR-944, ΔNp63 siRNA or negative control. Data are reported as means ± SD for five independent experiments and were analyzed using unpaired Mann-Whitney *U* test. ** *p* < 0.01.


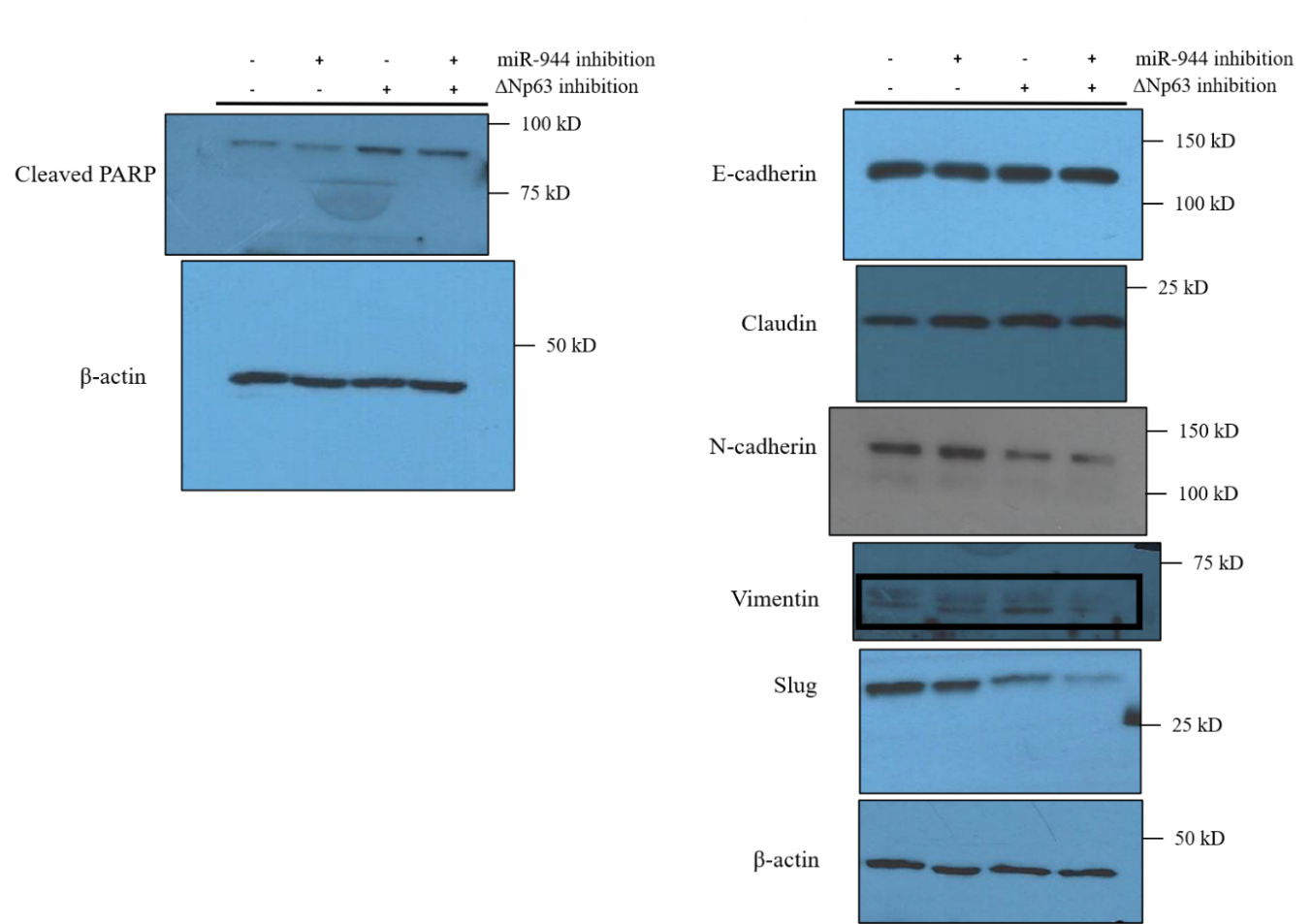


**Figure S2.** Uncropped original blots for western blot analysis**.**
